# Supplementary material for: ChatGPT as a Tool for Biostatisticians: A Tutorial on Applications, Opportunities, and Limitations
Source: Stat Med. 2025 Oct 23;44(23-24):e70263. doi: 10.1002/sim.70263 (PMC12548020; doi:10.1002/sim.70263)
Supplement: Supplementary file 1 — Data S1. Supporting Information. [file SIM-44-0-s001.zip › Supplementary Materials Additional.zip!/3.2_Diagnostic_Test/3.2_Diagnostic_Chat2.pdf]

ChatGPT - Diagnostic Testing

I would like to determine sensitivity and specificity of two diagnostic tests. I do have the diagnostic test outcomes, but I do not know the gold standard. What can I do?

Great question — and it touches on a real challenge in diagnostic test evaluation. In short: **if you don't know the true disease status (i.e. no gold standard), you cannot directly compute sensitivity or specificity**, because these require knowing who is truly diseased and who is not.

But — there *are* statistical methods to handle this situation. Here are some options:

1️⃣ Latent Class Analysis (LCA)

- This is the most common approach when there's no gold standard.
- Idea: treat the true disease status as a **latent (unobserved) variable**, and model the joint distribution of test results to infer both:
  - the prevalence of disease
  - sensitivity & specificity of each test
- Assumes **conditional independence** between tests given true disease status (though extensions exist if this assumption is violated).
- Available in software:
  - R packages: `poLCA`, `randomLCA`, `BayesLCA`, `lcmm`
  - Bayesian: `JAGS`, `Stan`

2️⃣ Bayesian Latent Class Models

- A Bayesian version of LCA:
  - You specify prior beliefs about sensitivity/specificity.
  - The model combines priors with observed test data to estimate posterior distributions of sensitivity, specificity, and prevalence.
- Can handle **correlated tests** and small sample sizes better than classical LCA.
- Software:
  - R + JAGS or Stan
  - `brms` package in R

3️⃣ Use an Imperfect Reference Standard

- If you have some reference test that is imperfect, you can model this explicitly:

- Adjust your estimates to account for the known sensitivity/specificity of the reference test.
- Example: "Imperfect gold standard" methods, or correction formulas.

4️⃣ Composite Reference Standard

- Define disease based on a combination of test results or clinical criteria.
- Can introduce bias but sometimes is a practical workaround.

Summary

Method	What it does	Pros	Cons
Latent Class Analysis	Estimates test performance without gold standard	Well-established	Conditional independence assumption
Bayesian Latent Class	Same as LCA, adds priors	Flexible, can handle dependencies	Requires Bayesian expertise
Imperfect Reference	Adjusts for imperfect gold standard	Simple	Need to know reference performance
Composite Standard	Combines tests/criteria	Easy to implement	May introduce bias

If you tell me:

1. How many tests you have
2. How large is your dataset
3. What type of tests (binary, continuous?)
4. If you can assume tests are independent

...I can suggest a more concrete path and even help write the code for you! Want to proceed? 🚀

Please, tell me more about the latent class analysis.

Of course — let's dive deeper into **Latent Class Analysis (LCA)** as applied to diagnostic tests. I'll explain in stages:

🔍 Concept

Imagine you have *n* patients and results from two (or more) diagnostic tests per patient.

The problem: You do not know whether each patient truly has the disease (no gold standard).

LCA idea:

- The "true disease status" is an *unobserved* or *latent* variable:

- Let's call it **D**: D = 1 (disease), D = 0 (no disease).
- Each test result is an *observed* variable:
    - Example: Test A (binary), Test B (binary).

LCA **models the joint probability** of the observed test results, assuming that this joint probability depends on the unobserved disease status.

🏗️ Model Structure

For **two binary tests** (A and B), here's the basic model:

Parameters:

- Prevalence of disease: **π = P(D = 1)**
- Sensitivity of Test A: **Se_A = P(Test A + | D = 1)**
- Specificity of Test A: **Sp_A = P(Test A − | D = 0)**
- Sensitivity of Test B: **Se_B**
- Specificity of Test B: **Sp_B**

Joint probabilities:

Each patient has one of 4 possible combinations of test outcomes:
(A+, B+), (A+, B−), (A−, B+), (A−, B−).

The probability of each combination is computed by:

$$P(\text{Test A outcome}, \text{Test B outcome}) = \pi \cdot P(\text{Test A outcome}|D=1) \cdot P(\text{Test B outcome}|D= [illegible]$$
$$+(1-\pi) \cdot P(\text{Test A outcome}|D=0) \cdot P(\text{Test B outcome}|D=0)$$

✨ Assumptions

Classic LCA makes these key assumptions:

1. **Conditional independence** of the tests *given* true disease status.
    - In other words: once you know D, the test results are independent.
2. Each test's sensitivity and specificity are constant across subjects.

Extensions exist to relax these assumptions.

🚀 Estimation

- You **fit the model** to the observed frequencies of test result combinations.
- The model finds the values of π, Se_A, Sp_A, Se_B, Sp_B that best explain the data.
- Methods:

- Maximum likelihood (EM algorithm)
- Bayesian estimation (MCMC)

🖥️ Example in R (2 tests, binary)

```r
# Example data: counts of combinations of test results
# A+, B+ | A+, B- | A-, B+ | A-, B-
obs_counts <- c(50, 30, 20, 100)

# Load poLCA package
library(poLCA)

# Format data: each test result as a column
df <- data.frame(
  TestA = c(rep(1, 50), rep(1, 30), rep(0, 20), rep(0, 100)),
  TestB = c(rep(1, 50), rep(0, 30), rep(1, 20), rep(0, 100))
)

# LCA model with 2 latent classes (disease / no disease)
lca_model <- poLCA(cbind(TestA, TestB) ~ 1, data=df, nclass=2)

# Outputs:
# - Prevalence (class probabilities)
# - Conditional probabilities (which give Se and Sp)
```

🧠 Interpretation

- The estimated **class prevalences** give disease prevalence.
- The **conditional probabilities** of each test result *within each class* give estimated sensitivities & specificities.

⚠️ Limitations

1. **Conditional independence** is often violated — tests may be correlated even within diseased/non-diseased patients.
   - Extensions (latent class with random effects, direct modeling of dependencies) are possible.
2. **Model identifiability**:
   - If you have only 2 tests, sometimes the model is not identifiable or unstable.
   - More tests (3+) generally improve identifiability and stability.
3. **Sample size**:
   - LCA works best with moderate to large samples.

🚩 Summary

Strengths	Weaknesses
No need for gold standard	Sensitive to model assumptions
Estimates Se, Sp & prevalence	Conditional independence may not hold

Strengths	Weaknesses
Flexible extensions available	Model identifiability can be tricky

If you tell me:

- how many tests you have (2? 3? more?)
- type of tests (binary / continuous)
- sample size

...I can show you an **exact example** for your case, with R code or Bayesian model if you prefer!

Want to proceed? 🚀

I have two populations with two different disease prevalences. I have two diagnostic tests which are conditional independent. I would like to get the log-likelihood of this setting.

Excellent — now you are describing a **two-population latent class model**, which is more identifiable than a one-population model! Let me walk you through the log-likelihood for this setting.

🚩 Your setting

- Two populations: indexed by **j = 1, 2**
    - Population 1: disease prevalence π_1
    - Population 2: disease prevalence π_2
- Two diagnostic tests: indexed **A** and **B**
    - Conditional independence given disease status
    - Sensitivity & specificity:
        - **Se_A**, **Sp_A** for test A
        - **Se_B**, **Sp_B** for test B

🏗️ Observed data

For each population, you observe counts of the 4 possible combinations of test results:

Test A	Test B	Count in Population 1	Count in Population 2
+	+	n_{111}	n_{211}
+	−	n_{110}	n_{210}
−	+	n_{101}	n_{201}
−	−	n_{100}	n_{200}

🔢 Model: Probabilities of each test combination

For population j (j = 1 or 2):

Let's define:

- π_j = disease prevalence in population j
- **D = 1** → diseased
- **D = 0** → non-diseased

Conditional probabilities of test results given disease status:

Test result	D = 1 (diseased)	D = 0 (non-diseased)
A +	**Se_A**	**1 − Sp_A**
A −	**1 − Se_A**	**Sp_A**
B +	**Se_B**	**1 − Sp_B**
B −	**1 − Se_B**	**Sp_B**

Marginal probability of each test result combination:

For a given population **j**, the probability of observing test result **(a, b)** is:

$$P_j(a,b) = \pi_j \cdot P(a|D=1) \cdot P(b|D=1) + (1-\pi_j) \cdot P(a|D=0) \cdot P(b|D=0)$$

where:

- **a = 1** → A+
- **a = 0** → A−
- **b = 1** → B+
- **b = 0** → B−

📜 Log-likelihood

The **total log-likelihood** is simply the sum over the two populations and the 4 test result combinations:

$$\log L = \sum_{j=1}^{2} \sum_{a=0}^{1} \sum_{b=0}^{1} n_{jab} \cdot \log P_j(a,b)$$

⚙️ Full expression

Putting it all together:

$$\log L = n_{111} \log P_1(1,1) + n_{110} \log P_1(1,0) + n_{101} \log P_1(0,1) + n_{100} \log P_1(0,0)$$
$$+ n_{211} \log P_2(1,1) + n_{210} \log P_2(1,0) + n_{201} \log P_2(0,1) + n_{200} \log P_2(0,0)$$

🚀 Summary of parameters

Parameter	Meaning
**π_1**	Prevalence in population 1
**π_2**	Prevalence in population 2
**Se_A**	Sensitivity of test A
**Sp_A**	Specificity of test A
**Se_B**	Sensitivity of test B
**Sp_B**	Specificity of test B

🖥️ R / Python implementation

You can easily implement this likelihood in R, Python, or any optimization software and then **maximize it** to estimate the parameters.

⚠️ Important

👉 **This model is identifiable** because you have:

- two populations with different prevalences,
- two conditionally independent tests.

🎁 If you want, I can provide:

✅ Full R code
✅ Full Python code
✅ Example with simulated data
✅ Extension to Bayesian model (with priors)

Would you like me to generate a **concrete example** for you — in R or Python? Just tell me which language you prefer 🚀.

What is the Hui-Walter paradigm?

Great follow-up question — the **Hui-Walter paradigm** is exactly the framework you've been describing! Let me explain it clearly:

🌟 Hui-Walter paradigm (1980)

The **Hui-Walter model** is the classic latent class model for estimating diagnostic test accuracy **without a gold standard**.

It was first proposed in:

📖 **Hui, S.L. & Walter, S.D. (1980). Estimating the Error Rates of Diagnostic Tests. Biometrics 36(1): 167–171.**

🚩 Key idea

Problem:
You have diagnostic tests, but no gold standard to tell you which individuals truly have disease.

Solution (Hui-Walter paradigm):
If you have:

1. **≥ 2 populations** with **different disease prevalences**
2. **≥ 2 conditionally independent tests**
   → Then, you can **identify** (i.e. estimate) test sensitivities, specificities, and prevalences using the observed data alone.

🏗️ Assumptions

1️⃣ There are **k ≥ 2 populations**
- Populations differ in disease prevalence: $\pi_1, \pi_2, \ldots, \pi_k$

2️⃣ There are **m ≥ 2 diagnostic tests**
- Tests are **conditionally independent given true disease status** (though this can be relaxed in extensions)

3️⃣ **Test characteristics (Se, Sp) are constant across populations**
- Sensitivity and specificity of each test do not change between populations

🔢 Why it works

- You observe the **joint test results** in each population.
- Because the populations have **different prevalences**, the joint distribution of test results **varies across populations**.
- This variation allows you to **separate** the effects of test sensitivity/specificity from disease prevalence.

🎓 Classic example: 2 populations, 2 tests

Parameter	Meaning
π_1, π_2	Prevalence in populations 1 and 2
Se_1, Sp_1	Sensitivity & specificity of Test 1
Se_2, Sp_2	Sensitivity & specificity of Test 2

Observed data:

For each population, you count the **4 combinations of test results**: (+,+), (+,−), (−,+), (−,−).

- You have **2 populations × 4 combinations = 8 observed counts**.
- You have **6 unknown parameters**:
  - π_1, π_2, Se_1, Sp_1, Se_2, Sp_2.

→ **The model is identifiable!**
(8 data points > 6 unknowns → you can estimate everything).

✨ Why it's famous

Before Hui & Walter, many people thought you **needed a gold standard** to estimate sensitivity/specificity.

Their key insight:
If you have **≥ 2 populations with different prevalences**, and **conditionally independent tests**, you can solve the model without knowing the true disease status of any individual.

⚠️ Limitations

Limitation	Comment
Conditional independence assumption	Often violated in practice (tests can be correlated even after conditioning on disease status)
Constant Se/Sp across populations	May not hold if patient characteristics differ a lot between populations
Requires ≥ 2 populations with different prevalences	Cannot apply in a single homogeneous population

🏆 Extensions

Modern latent class models extend the Hui-Walter paradigm to:
✅ More than 2 tests
✅ More than 2 populations
✅ Models with **dependent tests**
✅ **Bayesian** latent class models (with priors)
✅ **Random effects** for Se/Sp across populations

🚀 Summary

Classic Hui-Walter paradigm
≥ 2 populations with different prevalences
≥ 2 conditionally independent tests
Constant Se/Sp across populations
No gold standard required
Fully identifiable model

🎁 If you want, I can:

✅ Show you the **exact likelihood formula** for the Hui-Walter model
✅ Provide **R code** or **Python code** to fit it
✅ Show how to implement a **Bayesian version** with priors

Would you like me to generate an example Hui-Walter model for you?
👉 Just tell me:

1. **R** or **Python**?
2. Do you want **frequentist** (MLE) or **Bayesian** version?

Ready when you are 🚀.

I would like to see a worked example and a R script that implements this model.

Perfect! Let's do a full **worked example** of the classic **Hui-Walter paradigm** with **2 populations and 2 tests**, and I'll give you a ready-to-use **R script**.

🔍 Step 1: Simulate example data

Let's imagine:

- **Test A**: Se_A = 0.90, Sp_A = 0.95
- **Test B**: Se_B = 0.85, Sp_B = 0.92
- **Population 1**: prevalence π_1 = 0.60
- **Population 2**: prevalence π_2 = 0.20

We will simulate counts of all 4 combinations of test results in both populations.

🏗️ Step 2: Hui-Walter likelihood

We will implement the **log-likelihood** and use `optim()` to find MLE estimates of:

- π_1, π_2, Se_A, Sp_A, Se_B, Sp_B

🎁 Full R script

```r
# Load necessary library
library(tidyverse)

# Set true parameters
Se_A <- 0.90
Sp_A <- 0.95
```

```
Se_B <- 0.85
Sp_B <- 0.92
pi1  <- 0.60
pi2  <- 0.20

# Function to compute probabilities of test results
get_probs <- function(pi, Se_A, Sp_A, Se_B, Sp_B) {
  probs <- matrix(0, nrow=2, ncol=2)

  # P(A+, B+)
  probs[1,1] <- pi * Se_A * Se_B + (1 - pi) * (1 - Sp_A) * (1 - Sp_B)
  # P(A+, B-)
  probs[1,2] <- pi * Se_A * (1 - Se_B) + (1 - pi) * (1 - Sp_A) * Sp_B
  # P(A-, B+)
  probs[2,1] <- pi * (1 - Se_A) * Se_B + (1 - pi) * Sp_A * (1 - Sp_B)
  # P(A-, B-)
  probs[2,2] <- pi * (1 - Se_A) * (1 - Se_B) + (1 - pi) * Sp_A * Sp_B

  return(probs)
}

# Simulate data
set.seed(123)

# Sample sizes per population
N1 <- 1000
N2 <- 1000

# Population 1
probs1 <- get_probs(pi1, Se_A, Sp_A, Se_B, Sp_B)
counts1 <- as.vector(rmultinom(1, N1, prob = as.vector(probs1)))

# Population 2
probs2 <- get_probs(pi2, Se_A, Sp_A, Se_B, Sp_B)
counts2 <- as.vector(rmultinom(1, N2, prob = as.vector(probs2)))

# Show the counts
cat("Observed counts (Population 1):\n")
matrix(counts1, 2, 2)
cat("\nObserved counts (Population 2):\n")
matrix(counts2, 2, 2)

# Combine data
counts <- c(counts1, counts2)

# Log-likelihood function
loglik <- function(par, counts) {
  # Unpack parameters (logit scale for stability)
  pi1 <- plogis(par[1])
  pi2 <- plogis(par[2])
  Se_A <- plogis(par[3])
  Sp_A <- plogis(par[4])
  Se_B <- plogis(par[5])
  Sp_B <- plogis(par[6])

  # Probabilities for each population
  p1 <- get_probs(pi1, Se_A, Sp_A, Se_B, Sp_B)
  p2 <- get_probs(pi2, Se_A, Sp_A, Se_B, Sp_B)

  # Log-likelihood
  ll <- 0
  ll <- ll + sum(counts[1:4] * log(as.vector(p1)))
  ll <- ll + sum(counts[5:8] * log(as.vector(p2)))

  return(-ll)  # Negative for minimization
}

# Initial values (logit scale)
init <- qlogis(c(0.5, 0.5, 0.8, 0.8, 0.8, 0.8))

# Run optimization
fit <- optim(init, loglik, counts = counts, method = "BFGS", hessian = TRUE)

# Transform back to original scale
est <- plogis(fit$par)
```

```
names(est) <- c("pi1", "pi2", "Se_A", "Sp_A", "Se_B", "Sp_B")

# Show results
cat("\nEstimated parameters:\n")
print(est)

# True values for comparison
cat("\nTrue values:\n")
true_vals <- c(pi1, pi2, Se_A, Sp_A, Se_B, Sp_B)
names(true_vals) <- names(est)
print(true_vals)
```

🚀 How to run it

1️⃣ Copy the script into an R file (e.g. **`hui_walter_example.R`**)
2️⃣ Run it in RStudio or R console
3️⃣ It will print:

- The simulated observed counts
- The estimated parameters (MLE)
- The true values (for comparison)

🎓 What you will see

Example output might look like:

```
less

Observed counts (Population 1):
      [,1] [,2]
[1,]   457   47
[2,]    78  418

Observed counts (Population 2):
      [,1] [,2]
[1,]   117   23
[2,]    59  801

Estimated parameters:
    pi1      pi2      Se_A     Sp_A     Se_B     Sp_B
0.5987   0.2031   0.9021   0.9492   0.8514   0.9187

True values:
    pi1      pi2      Se_A     Sp_A     Se_B     Sp_B
0.6000   0.2000   0.9000   0.9500   0.8500   0.9200
```

⚠️ Interpretation

✅ The **estimated parameters** should be very close to the **true values**, showing that the **Hui-Walter model works**!
✅ You didn't need a gold standard — just **2 populations** with different prevalences and **2 tests**.

🎁 Possible extensions

- More than 2 populations

- More than 2 tests
- Bayesian version (with priors)
- Model with test dependence (correlation between tests)

🎯 Summary

Concept	This example
**Hui-Walter model**	Yes
**2 populations**	Yes
**2 tests**	Yes
**Conditional independence**	Yes
**Maximum likelihood estimation**	Yes
**R script provided**	Yes

If you like, I can also generate:
✅ A **Bayesian version** of this model (using `rjags` or `brms`)
✅ A version with **3 tests**
✅ A version with **correlated tests**

Would you like me to show you one of these next? 🚀

I would like to add confidence intervals to the estimated sensitivities, specificities, and prevalences.

Excellent — very good idea!

You already noticed that **optim()** gives point estimates, but for **confidence intervals (CIs)** you need to compute uncertainty, typically using the **variance-covariance matrix** of the MLEs.

🚀 Here's how to do it:

1️⃣ The `optim()` function with `hessian=TRUE` returns the **Hessian matrix** of the log-likelihood at the MLE.

2️⃣ The **inverse of the Hessian** is an estimate of the **variance-covariance matrix** of the parameters — on the **logit scale** (because we used logit transforms).

3️⃣ To compute CIs:

- Compute **standard errors (SEs)** on the logit scale.
- Compute **CIs** on the logit scale.
- **Back-transform** to the original scale (probability scale) using the logistic function.

🎁 Updated R code snippet for CIs

After your `optim()` call:

```r
# Inverse Hessian → Variance-covariance matrix (on logit scale)
vcov_mat <- solve(fit$hessian)

# Standard errors (on logit scale)
se_logit <- sqrt(diag(vcov_mat))

# 95% CI on logit scale: estimate ± 1.96 * SE
lower_logit <- fit$par - 1.96 * se_logit
upper_logit <- fit$par + 1.96 * se_logit

# Back-transform to probability scale
estimate_prob <- plogis(fit$par)
lower_prob   <- plogis(lower_logit)
upper_prob   <- plogis(upper_logit)

# Combine results
results <- data.frame(
  Parameter = c("pi1", "pi2", "Se_A", "Sp_A", "Se_B", "Sp_B"),
  Estimate = estimate_prob,
  Lower_95_CI = lower_prob,
  Upper_95_CI = upper_prob
)

# Print nicely
cat("\nEstimated parameters with 95% confidence intervals:\n")
print(results)
```

⚠️ Interpretation

✅ The CIs reflect the **sampling uncertainty** of your MLE estimates.
✅ If sample size is large, CIs will be narrow.
✅ If tests are nearly collinear or populations are too similar, CIs will be wide (possible identifiability problems).

🎓 Summary

Step	Method
Point estimates	from **`optim()`** MLE
Variance-covariance matrix	inverse Hessian
Confidence intervals	logit scale → back-transform

✨ Full flow

If you want, I can post an **updated full script** with this built-in — so you can just copy & run one complete file.
Would you like me to generate the full version for you now? 🚀

I would like to run a bootstrap version for robust confidence intervals.

Excellent idea! 🎓
Bootstrap CIs are **robust** and don't rely on the Hessian approximation or normality of MLEs — perfect for latent class models, which can be a bit "non-regular" in small samples.

🔍 How does bootstrap work here?

You will:
1️⃣ **Resample** patients *within each population* (with replacement).
→ This preserves the prevalence differences between populations.

2️⃣ For each bootstrap sample:
- Recalculate the **counts of test result combinations**.
- Refit the model (optimize log-likelihood).
- Save the estimated parameters.

3️⃣ After many bootstrap replicates (say 1000), you:
- Take empirical **percentiles** of the bootstrap estimates to form **95% CIs**.

🚀 Bootstrap version — template R code

Below I show you how to modify your script to add **bootstrap**:

```r
# Load necessary library
library(tidyverse)

# How many bootstrap replicates?
n_boot <- 1000

# Storage for bootstrap estimates
boot_estimates <- matrix(NA, nrow = n_boot, ncol = 6)
colnames(boot_estimates) <- c("pi1", "pi2", "Se_A", "Sp_A", "Se_B", "Sp_B")

# Function to run one optimization
fit_model <- function(counts) {
  # Initial values
  init <- qlogis(c(0.5, 0.5, 0.8, 0.8, 0.8, 0.8))

  # Optimize
  fit <- optim(init, loglik, counts = counts, method = "BFGS")

  # Return estimated parameters (probability scale)
  return(plogis(fit$par))
}

# Original data (you can reuse from previous simulation)
# For demonstration, let's assume you already have:
# - N1, N2: sample sizes
# - probs1, probs2: true probabilities
# We resimulate the original full individual-level data here:

# Simulate individual-level data
sim_data <- function(N, probs) {
  test_combos <- expand.grid(A = c(1, 0), B = c(1, 0))
  sample_idx <- sample(1:4, size = N, replace = TRUE, prob = as.vector(probs))
  df <- test_combos[sample_idx, ]
  return(df)
}

# Original sample:
df1_orig <- sim_data(N1, probs1)
```

```r
df2_orig <- sim_data(N2, probs2)

# Bootstrap loop
set.seed(12345)
pb <- txtProgressBar(min = 0, max = n_boot, style = 3)

for (b in 1:n_boot) {
  # Resample within each population
  df1_boot <- df1_orig[sample(1:N1, N1, replace = TRUE), ]
  df2_boot <- df2_orig[sample(1:N2, N2, replace = TRUE), ]

  # Tabulate counts
  counts1 <- table(factor(df1_boot$A, levels = c(1,0)),
                   factor(df1_boot$B, levels = c(1,0)))
  counts2 <- table(factor(df2_boot$A, levels = c(1,0)),
                   factor(df2_boot$B, levels = c(1,0)))

  # Vectorize counts
  counts_boot <- c(as.vector(counts1), as.vector(counts2))

  # Fit model
  est <- tryCatch({
    fit_model(counts_boot)
  }, error = function(e) rep(NA, 6))  # In case of failed optimization

  # Store
  boot_estimates[b, ] <- est

  setTxtProgressBar(pb, b)
}
close(pb)

# Remove failed runs
boot_estimates <- boot_estimates[complete.cases(boot_estimates), ]

# Summary: bootstrap 95% CIs (percentile method)
boot_CI <- apply(boot_estimates, 2, quantile, probs = c(0.025, 0.975))

# Show results
cat("\nBootstrap 95% confidence intervals:\n")
print(t(boot_CI))
```

✨ Interpretation

What you get	Meaning
**`boot_estimates`**	All bootstrap parameter estimates
**`boot_CI`**	Robust 95% percentile CIs for each parameter

📜 Summary flow

1️⃣ Run your **original MLE** → point estimates
2️⃣ Run **bootstrap** → empirical CIs
3️⃣ Compare:
- Bootstrap CIs
- Hessian-based CIs (optional, already shown in previous answer)

⚠️ Notes

✅ Bootstrap is **more robust** if:

- Sample size is moderate or small
- Model is somewhat complex
- The MLE is "irregular" (latent class models often are)

✅ But: Bootstrap is **computationally intensive** — be patient!

🎁 Next:

If you want, I can also give you:
✅ Full **integrated R script** with:

- Point estimates
- Hessian-based CIs
- Bootstrap CIs

✅ **Ready-to-run** in one file (easier for reproducibility)

Would you like me to generate this **full final version** for you?
Just say **yes** 🚀.

I would like to reparametrize my model using a logistic parametrization for the prevalences, sensitivities, and specificities.

Excellent — this is a very good modeling step!
You are asking for a **logistic reparametrization** — which is exactly what people do to ensure that the parameters stay in $[0, 1]$, and to improve numerical stability.

🎓 What does it mean?

You replace:

$$\pi_j = \text{plogis}(\theta_{\pi_j}) = \frac{1}{1 + e^{-\theta_{\pi_j}}}$$

$$\text{Se}_A = \text{plogis}(\theta_{\text{Se}_A})$$

$$\text{Sp}_A = \text{plogis}(\theta_{\text{Sp}_A})$$

$$\text{Se}_B = \text{plogis}(\theta_{\text{Se}_B})$$

$$\text{Sp}_B = \text{plogis}(\theta_{\text{Sp}_B})$$

🚀 Why do this?

✅ Now your optimization is over **unconstrained real parameters** $\theta \in (-\infty, +\infty)$,
✅ No need for bounds in optim(),
✅ Guarantees that on the probability scale everything stays in $[0, 1]$.

🔍 How to implement?

You already almost had this! You just need to "rename" the parameters to reflect this idea clearly:

🎁 Reparametrized log-likelihood

```r
loglik <- function(theta, counts) {
  # Reparametrize
  pi1 <- plogis(theta[1])
  pi2 <- plogis(theta[2])

  Se_A <- plogis(theta[3])
  Sp_A <- plogis(theta[4])

  Se_B <- plogis(theta[5])
  Sp_B <- plogis(theta[6])

  # Compute probabilities of test outcomes
  p1 <- get_probs(pi1, Se_A, Sp_A, Se_B, Sp_B)
  p2 <- get_probs(pi2, Se_A, Sp_A, Se_B, Sp_B)

  # Log-likelihood
  ll <- 0
  ll <- ll + sum(counts[1:4] * log(as.vector(p1)))
  ll <- ll + sum(counts[5:8] * log(as.vector(p2)))

  return(-ll)  # For minimization
}
```

🔢 Initial values

You choose initial values **on logit scale** (can be more flexible now):

```r
# Initial values (logit scale)
# Example: start at logit(0.5) for prevalences, logit(0.8) for Se/Sp
theta_init <- qlogis(c(0.5, 0.5, 0.8, 0.8, 0.8, 0.8))
```

🚀 Run optimization

```r
# Run optimization
fit <- optim(theta_init, loglik, counts = counts, method = "BFGS", hessian = TRUE)

# Transform back to probability scale
estimate_prob <- plogis(fit$par)
names(estimate_prob) <- c("pi1", "pi2", "Se_A", "Sp_A", "Se_B", "Sp_B")
```

```
# Show point estimates
print(estimate_prob)
```

⚠️ Why this is good practice

✅ Improves optimization stability
✅ Avoids boundary problems (Se or Sp near 0 or 1 can crash naive optim)
✅ Matches how Bayesian models (Stan, JAGS) handle these parameters
✅ Makes it trivial to add covariates later (via logistic regression on θ)

🚀 Summary

Original parameter	Reparametrized form
π_1, π_2	plogis(θ_π_1), plogis(θ_π_2)
Se_A, Sp_A	plogis(θ_Se_A), plogis(θ_Sp_A)
Se_B, Sp_B	plogis(θ_Se_B), plogis(θ_Sp_B)

🎁 Next step

✅ You can now also **bootstrap the θ's**
✅ Then **transform bootstrap θ's back to probability scale** → get robust CIs!
✅ Or run **Bayesian version** where priors are put on θ's — very natural.

Would you like me to generate:
1️⃣ Full **final script** (reparametrized, with bootstrap)?
2️⃣ Full **Bayesian version** (same parametrization)?

Just say **yes**, and tell me:

- Do you prefer **R frequentist** or **R Bayesian** for the next example 🚀.

Please update the bootstrap function too.

Excellent — let me show you exactly how to update the **bootstrap loop** to match the **logistic parametrization**.

The **good news**:
The *structure of the bootstrap* doesn't change — you still:
✅ resample within populations
✅ recompute counts
✅ refit the model → now using the **θ parameters** (logit scale)
✅ save results on **probability scale**

🚀 Updated bootstrap loop (with logistic parametrization)

```r
# --- Bootstrap parameters ---

n_boot <- 1000  # number of bootstrap replicates

# Storage matrix for bootstrap results (probability scale)
boot_estimates <- matrix(NA, nrow = n_boot, ncol = 6)
colnames(boot_estimates) <- c("pi1", "pi2", "Se_A", "Sp_A", "Se_B", "Sp_B")

# --- Helper: model fitting function ---

fit_model_logistic <- function(counts) {
  # Initial values on logit scale
  theta_init <- qlogis(c(0.5, 0.5, 0.8, 0.8, 0.8, 0.8))

  # Run optimization
  fit <- optim(theta_init, loglik, counts = counts, method = "BFGS")

  # Return estimates on probability scale
  return(plogis(fit$par))
}

# --- Bootstrap loop ---

set.seed(12345)
pb <- txtProgressBar(min = 0, max = n_boot, style = 3)

for (b in 1:n_boot) {

  # Resample within each population
  df1_boot <- df1_orig[sample(1:N1, N1, replace = TRUE), ]
  df2_boot <- df2_orig[sample(1:N2, N2, replace = TRUE), ]

  # Tabulate counts
  counts1 <- table(factor(df1_boot$A, levels = c(1, 0)),
                   factor(df1_boot$B, levels = c(1, 0)))

  counts2 <- table(factor(df2_boot$A, levels = c(1, 0)),
                   factor(df2_boot$B, levels = c(1, 0)))

  # Vectorize counts
  counts_boot <- c(as.vector(counts1), as.vector(counts2))

  # Fit model
  est <- tryCatch({
    fit_model_logistic(counts_boot)
  }, error = function(e) rep(NA, 6))  # if error, fill NA

  # Store bootstrap result
  boot_estimates[b, ] <- est

  # Progress bar update
  setTxtProgressBar(pb, b)
}

close(pb)

# --- Remove failed bootstrap runs ---
boot_estimates <- boot_estimates[complete.cases(boot_estimates), ]

# --- Summarize: Bootstrap 95% percentile CIs ---
boot_CI <- apply(boot_estimates, 2, quantile, probs = c(0.025, 0.975))

# --- Print results ---
cat("\nBootstrap 95% confidence intervals (percentile method):\n")
print(t(boot_CI))
```

⚠️ What changed from before?

Part	Change
Model fitting function	Now uses **fit_model_logistic()** → matches logistic parametrization
Saved estimates	Always save **probability scale** (plogis of θ) → matches main model output
Bootstrap loop	Structure same, just using logistic version

🚀 Summary workflow

✅ Main model → reparametrized using **θ** (logistic)
✅ Bootstrap → resample + **refit on θ** → back-transform → save
✅ Bootstrap CIs → based on **probability scale**

🎁 Final step: Integrated full script

If you'd like, I can generate for you a **single full script** that does:

✅ Reparametrized model
✅ Point estimates
✅ Hessian-based CIs
✅ Bootstrap CIs (robust)

→ Very clean, reproducible, ready to run.

Would you like me to generate this full version?
👉 Just say **yes** and I'll post it 🚀.

Could you also perform the calculation of the 95% Confidence intervals via the hessian?

Of course — that's an **essential step** for comparison!
I'll show you exactly how to do it in your **logistic reparametrization** version.

🎓 Key point

You already optimized **θ** (logit scale):

```r
fit <- optim(theta_init, loglik, counts = counts, method = "BFGS", hessian = TRUE)
```

✅ The **Hessian** is the second derivative of the log-likelihood w.r.t **θ**.
✅ The **inverse Hessian** is the **variance-covariance matrix** of **θ-hat** (on logit scale).
✅ Standard errors are √(diag of var-cov matrix).

Then you can compute **95% Wald-type CIs**:

$$\theta_i \pm 1.96 \times SE(\theta_i)$$

And back-transform using **plogis()** to probability scale.

🚀 Concrete R code

```r
# Inverse Hessian → variance-covariance matrix (on logit scale)
vcov_mat <- solve(fit$hessian)

# Standard errors on logit scale
se_logit <- sqrt(diag(vcov_mat))

# 95% CI on logit scale: θ ± 1.96 * SE
lower_logit <- fit$par - 1.96 * se_logit
upper_logit <- fit$par + 1.96 * se_logit

# Back-transform to probability scale
estimate_prob <- plogis(fit$par)
lower_prob    <- plogis(lower_logit)
upper_prob    <- plogis(upper_logit)

# Combine results
results_hessian_CI <- data.frame(
  Parameter   = c("pi1", "pi2", "Se_A", "Sp_A", "Se_B", "Sp_B"),
  Estimate    = estimate_prob,
  Lower_95_CI = lower_prob,
  Upper_95_CI = upper_prob
)

# Print
cat("\nHessian-based 95% confidence intervals (Wald method):\n")
print(results_hessian_CI)
```

⚠️ Notes

✅ These CIs are **asymptotic** — valid if sample size is big enough and MLE behaves well.
✅ They assume **normality on logit scale**.
✅ If the model is near boundaries (Se ≈ 1, Sp ≈ 1), **bootstrap CIs** are more reliable.

🚀 Summary

Step	What happens
**`fit$hessian`**	Hessian matrix at MLE (on θ scale)
**`solve(fit$hessian)`**	variance-covariance matrix of θ
SE = √(diag)	Standard errors of θ
θ ± 1.96 * SE	95% Wald CI on θ scale
plogis()	Back to probability scale

🎁 If you'd like:

✅ I can generate **the full final script**:
1️⃣ Logistic parametrization

2️⃣ Point estimates
3️⃣ Hessian-based CIs
4️⃣ Bootstrap CIs (robust)
→ Ready-to-run, very clean.

Would you like me to generate and post this **full, final version** for you? 🚀
Just say **yes**!

Please provide a comprehensive R code for the issues discussed in this session.

Perfect — here is a **comprehensive R script** that puts it **all together**:
✅ Logistic reparametrization
✅ Point estimates
✅ Hessian-based 95% CIs
✅ Bootstrap 95% CIs
✅ Clean and ready-to-run!

📜 Comprehensive Hui-Walter Example with Full Code

```r
# Hui-Walter paradigm: 2 populations, 2 tests
# Comprehensive R script with:
# - Logistic reparametrization
# - Point estimates
# - Hessian-based CIs
# - Bootstrap CIs

# --- Setup ---

library(tidyverse)

# --- Step 1: Simulate example data ---

# True parameters
Se_A <- 0.90
Sp_A <- 0.95
Se_B <- 0.85
Sp_B <- 0.92
pi1  <- 0.60
pi2  <- 0.20

# Function to compute P(test outcome combo | parameters)
get_probs <- function(pi, Se_A, Sp_A, Se_B, Sp_B) {
  probs <- matrix(0, nrow = 2, ncol = 2)

  probs[1,1] <- pi * Se_A * Se_B + (1 - pi) * (1 - Sp_A) * (1 - Sp_B)
  probs[1,2] <- pi * Se_A * (1 - Se_B) + (1 - pi) * (1 - Sp_A) * Sp_B
  probs[2,1] <- pi * (1 - Se_A) * Se_B + (1 - pi) * Sp_A * (1 - Sp_B)
  probs[2,2] <- pi * (1 - Se_A) * (1 - Se_B) + (1 - pi) * Sp_A * Sp_B

  return(probs)
}

# Simulate data
set.seed(123)
```

```r
N1 <- 1000
N2 <- 1000

probs1 <- get_probs(pi1, Se_A, Sp_A, Se_B, Sp_B)
probs2 <- get_probs(pi2, Se_A, Sp_A, Se_B, Sp_B)

counts1 <- as.vector(rmultinom(1, N1, prob = as.vector(probs1)))
counts2 <- as.vector(rmultinom(1, N2, prob = as.vector(probs2)))

counts <- c(counts1, counts2)

cat("Observed counts (Population 1):\n")
print(matrix(counts1, 2, 2))
cat("\nObserved counts (Population 2):\n")
print(matrix(counts2, 2, 2))

# --- Step 2: Define logistic log-likelihood ---

loglik <- function(theta, counts) {
  pi1 <- plogis(theta[1])
  pi2 <- plogis(theta[2])
  Se_A <- plogis(theta[3])
  Sp_A <- plogis(theta[4])
  Se_B <- plogis(theta[5])
  Sp_B <- plogis(theta[6])

  p1 <- get_probs(pi1, Se_A, Sp_A, Se_B, Sp_B)
  p2 <- get_probs(pi2, Se_A, Sp_A, Se_B, Sp_B)

  ll <- sum(counts[1:4] * log(as.vector(p1))) +
        sum(counts[5:8] * log(as.vector(p2)))

  return(-ll)  # for minimization
}

# --- Step 3: MLE estimation ---

theta_init <- qlogis(c(0.5, 0.5, 0.8, 0.8, 0.8, 0.8))

fit <- optim(theta_init, loglik, counts = counts, method = "BFGS", hessian = TRUE)

estimate_prob <- plogis(fit$par)
names(estimate_prob) <- c("pi1", "pi2", "Se_A", "Sp_A", "Se_B", "Sp_B")

cat("\n--- Point estimates (MLE): ---\n")
print(estimate_prob)

# --- Step 4: Hessian-based 95% CIs ---

vcov_mat <- solve(fit$hessian)
se_logit <- sqrt(diag(vcov_mat))

lower_logit <- fit$par - 1.96 * se_logit
upper_logit <- fit$par + 1.96 * se_logit

lower_prob <- plogis(lower_logit)
upper_prob <- plogis(upper_logit)

results_hessian_CI <- data.frame(
  Parameter = names(estimate_prob),
  Estimate = estimate_prob,
  Lower_95_CI = lower_prob,
  Upper_95_CI = upper_prob
)

cat("\n--- Hessian-based 95% CIs (Wald): ---\n")
print(results_hessian_CI)

# --- Step 5: Bootstrap 95% CIs ---

# Simulate individual-level data for bootstrap
sim_data <- function(N, probs) {
  test_combos <- expand.grid(A = c(1, 0), B = c(1, 0))
  sample_idx <- sample(1:4, size = N, replace = TRUE, prob = as.vector(probs))
  df <- test_combos[sample_idx, ]
```

```
  return(df)
}

df1_orig <- sim_data(N1, probs1)
df2_orig <- sim_data(N2, probs2)

# Helper function to fit model
fit_model_logistic <- function(counts) {
  theta_init <- qlogis(c(0.5, 0.5, 0.8, 0.8, 0.8, 0.8))
  fit <- optim(theta_init, loglik, counts = counts, method = "BFGS")
  return(plogis(fit$par))
}

# Bootstrap loop
n_boot <- 1000
boot_estimates <- matrix(NA, nrow = n_boot, ncol = 6)
colnames(boot_estimates) <- names(estimate_prob)

set.seed(12345)
pb <- txtProgressBar(min = 0, max = n_boot, style = 3)

for (b in 1:n_boot) {
  df1_boot <- df1_orig[sample(1:N1, N1, replace = TRUE), ]
  df2_boot <- df2_orig[sample(1:N2, N2, replace = TRUE), ]

  counts1 <- table(factor(df1_boot$A, levels = c(1, 0)),
                   factor(df1_boot$B, levels = c(1, 0)))
  counts2 <- table(factor(df2_boot$A, levels = c(1, 0)),
                   factor(df2_boot$B, levels = c(1, 0)))

  counts_boot <- c(as.vector(counts1), as.vector(counts2))

  est <- tryCatch({
    fit_model_logistic(counts_boot)
  }, error = function(e) rep(NA, 6))

  boot_estimates[b, ] <- est
  setTxtProgressBar(pb, b)
}

close(pb)

boot_estimates <- boot_estimates[complete.cases(boot_estimates), ]

boot_CI <- apply(boot_estimates, 2, quantile, probs = c(0.025, 0.975))

cat("\n--- Bootstrap 95% CIs (percentile): ---\n")
print(t(boot_CI))

# --- Compare to true values ---

true_vals <- c(pi1, pi2, Se_A, Sp_A, Se_B, Sp_B)
names(true_vals) <- names(estimate_prob)

cat("\n--- True parameter values: ---\n")
print(true_vals)
```

🎯 What this script does

✅ Simulates realistic data (you can replace this with your real data)
✅ MLE point estimates (with logistic reparametrization)
✅ Hessian-based 95% CIs
✅ Bootstrap 95% CIs (robust)
✅ Progress bar for bootstrap
✅ Prints everything nicely for interpretation

🚀 How to use it

1️⃣ Save this as `hui_walter_full.R`
2️⃣ Run in RStudio or R console
3️⃣ You will see:

- Observed counts
- MLE estimates
- Hessian CIs
- Bootstrap CIs
- Comparison to true values (for validation)

🎁 Possible next extensions

✅ Bayesian version (with priors) — very natural with this parametrization
✅ Add more than 2 tests
✅ Add more than 2 populations
✅ Model test dependence (correlation between tests)

If you want, I can also generate for you:
✅ **Bayesian version in R** using `rjags` or `brms`
✅ **Stan** version (very fast and elegant)

Would you like me to generate the **Bayesian version** next?
Just say **yes** 🚀.
